# Recommendations on data sharing in HIV drug resistance research

**DOI:** 10.1371/journal.pmed.1004293

**Published:** 2023-09-22

**Authors:** Seth C. Inzaule, Mark J. Siedner, Susan J. Little, Santiago Avila-Rios, Alisen Ayitewala, Ronald J. Bosch, Vincent Calvez, Francesca Ceccherini-Silberstein, Charlotte Charpentier, Diane Descamps, Susan H. Eshleman, Joseph Fokam, Lisa M. Frenkel, Ravindra K. Gupta, John P.A. Ioannidis, Pontiano Kaleebu, Rami Kantor, Seble G. Kassaye, Sergei L. Kosakovsky Pond, Vinie Kouamou, Roger D. Kouyos, Daniel R. Kuritzkes, Richard Lessells, Anne-Genevieve Marcelin, Lawrence Mbuagbaw, Brian Minalga, Nicaise Ndembi, Richard A. Neher, Roger Paredes, Deenan Pillay, Elliot G. Raizes, Soo-Yon Rhee, Douglas D. Richman, Kiat Ruxrungtham, Pardis C. Sabeti, Jonathan M. Schapiro, Sunee Sirivichayakul, Kim Steegen, Wataru Sugiura, Gert U. van Zyl, Anne-Mieke Vandamme, Annemarie M.J. Wensing, Joel O. Wertheim, Huldrych F. Gunthard, Michael R. Jordan, Robert W. Shafer

**Affiliations:** 1 Amsterdam Institute for Global Health and Development, and Department of Global Health, Amsterdam UMC, University of Amsterdam, Amsterdam, the Netherlands; 2 Department of Medicine, Harvard Medical School, Boston, Massachusetts, United States of America; 3 Division of Infectious Diseases and Global Public Health, Department of Medicine, University of California San Diego, San Diego, California, United States of America; 4 Centre for Research in Infectious Diseases, National Institute of Respiratory Diseases, Mexico City, Mexico; 5 National Health Laboratories and Diagnostic Services, Ministries of Health, Kampala, Uganda; 6 Center for Biostatistics in AIDS Research, Harvard TH Chan School of Public Health, Boston, Massachusetts, United States of America; 7 Sorbonne Université, INSERM, Institut Pierre Louis d’Epidémiologie et de Santé Publique (iPLESP), AP-HP, Hôpital Pitié-Salpêtrière, Service de Virologie, Paris, France; 8 Department of Experimental Medicine, University of Rome Tor Vergata, Rome, Italy; 9 Service de Virologie, Université Paris Cité, INSERM, IAME, UMR 1137, AP-HP, Hôpital Bichat-Claude Bernard, F-75018 Paris, France; 10 Department of Pathology, Johns Hopkins University School of Medicine, Baltimore, Maryland, United States of America; 11 Virology Laboratory, Chantal BIYA International Reference Centre for Research on HIV/AIDS Prevention and Management, Yaoundé, Cameroon, and Faculty of Health Sciences, University of Buea, Yaoundé, Cameroon; 12 Department of Pediatrics, University of Washington, Seattle, Washington, United States of America; 13 Cambridge Institute of Therapeutic Immunology and Infectious Diseases, University of Cambridge, Cambridge, United Kingdom; 14 Department of Medicine, Department of Epidemiology and Population Health, and Meta-Research Innovation Center at Stanford (METRICS), Stanford University, Stanford, California, United States of America; 15 Medical Research Council/Uganda Virus Research Institute and London School of Hygiene and Tropical Medicine, Entebbe, Uganda; 16 Department of Medicine, Brown University, The Miriam Hospital, Providence, Rhode Island, United States of America; 17 Department of Medicine, Division of Infectious Diseases, Georgetown University, Washington DC, United States of America; 18 Institute for Genomics and Evolutionary Medicine, Temple University, Philadelphia, Pennsylvania, United States of America; 19 Faculty of Medicine and Health Sciences, University of Zimbabwe, Harare, Zimbabwe; 20 Department of Infectious Diseases and Hospital Epidemiology, University Hospital Zurich, Zurich, Switzerland and Institute of Medical Virology, University of Zurich, Zurich, Switzerland; 21 Division of Infectious Diseases, Department of Medicine, Brigham and Women’s Hospital, Harvard Medical School, Boston, Massachusetts, United States of America; 22 Affiliation is KwaZulu-Natal Research Innovation & Sequencing Platform, University of KwaZulu-Natal, Durban, South Africa; 23 Sorbonne Université, INSERM, Institut Pierre Louis d’Epidémiologie et de Santé Publique, AP-HP, Hôpitaux Universitaires Pitié-Salpêtrière—Charles Foix, Laboratoire de Virologie, Paris, France; 24 Department of Health Research Methods, Evidence, and Impact, McMaster University, Hamilton, Canada; 25 Office of HIV/AIDS Network Coordination, Fred Hutchinson Cancer Center, Seattle, Washington, United States of America; 26 Institute of Human Virology Nigeria, Herbert Macaulay Way, Abuja, Nigeria; 27 Biozentrum, University of Basel, Basel, Switzerland; 28 Department of Infectious Diseases & irsiCaixa, Hospital Universitari Germans Trias i Pujol, Badalona, Catalonia, Spain; 29 Division of Infection and Immunity, University College London, London, United Kingdom; 30 United States Centers for Disease Control and Prevention, Atlanta, Georgia, United States of America; 31 Division of Infectious Diseases, Department of Medicine, Stanford University, Stanford, California, United States of America; 32 Center for AIDS Research, Department of Medicine, University of California San Diego, San Diego, California, United States of America; 33 School of Global Health, Faculty of Medicine, Chulalongkorn University, Bangkok, Thailand; 34 Broad Institute of MIT and Harvard, Cambridge, Massachusetts, United States of America; 35 National Hemophilia Center, Sheba Medical Center, Ramat Gan, Israel; 36 Faculty of Medicine, Chulalongkorn University, Bangkok, Thailand; 37 Department of Molecular Medicine and Haematology, National Health Laboratory Service, Johannesburg, South Africa; 38 Center for Clinical Sciences, National Center for Global Health and Medicine, Tokyo, Japan; 39 Division of Medical Virology, Stellenbosch University and National Health Laboratory Service, Cape Town, South Africa; 40 KU Leuven, Department of Microbiology, Immunology and Transplantation, Rega Institute for Medical Research, Clinical and Epidemiological Virology, Leuven, Belgium; 41 Center for Global Health And Tropical Medicine, Instituto de Higiene e Medicina Tropical, Universidade Nova de Lisboa, Lisbon, Portugal; 42 University Medical Center Utrecht, the Netherlands and Wits Reproductive Health and HIV Institute, University of the Witwatersrand, Johannesburg, South Africa; 43 Department of Medicine, University of California San Diego, San Diego, La Jolla, California, United States of America; 44 Department of Medicine, Tufts University School of Medicine, Boston, Massachusetts, United States of America

## Abstract

• Human immunodeficiency virus (HIV) drug resistance has implications for antiretroviral treatment strategies and for containing the HIV pandemic because the development of HIV drug resistance leads to the requirement for antiretroviral drugs that may be less effective, less well-tolerated, and more expensive than those used in first-line regimens.

• HIV drug resistance studies are designed to determine which HIV mutations are selected by antiretroviral drugs and, in turn, how these mutations affect antiretroviral drug susceptibility and response to future antiretroviral treatment regimens.

• Such studies collectively form a vital knowledge base essential for monitoring global HIV drug resistance trends, interpreting HIV genotypic tests, and updating HIV treatment guidelines.

• Although HIV drug resistance data are collected in many studies, such data are often not publicly shared, prompting the need to recommend best practices to encourage and standardize HIV drug resistance data sharing.

• In contrast to other viruses, sharing HIV sequences from phylogenetic studies of transmission dynamics requires additional precautions as HIV transmission is criminalized in many countries and regions.

• Our recommendations are designed to ensure that the data that contribute to HIV drug resistance knowledge will be available without undue hardship to those publishing HIV drug resistance studies and without risk to people living with HIV.

## Introduction

Data sharing can be beneficial to many stakeholders in biomedical research, including researchers, regulatory and funding agencies, and the public. It enables the reproduction and validation of studies and facilitates the consolidation of data from multiple studies to address common questions unanswerable by a single study. Many scientific organizations and funding agencies mandate that scientists incorporate data-sharing plans in their funding applications, protocols, and manuscripts. In 2014, the National Institutes Genomic Data Sharing Policy recommended that investigators generating genomic data seek consent from participants for the broadest possible sharing of research data [1]. In 2020, the US National Institutes of Health (NIH) drafted new guidelines for data management and sharing, which took effect in January 2023 [2]. In 2022, the World Health Organization (WHO) issued a policy promoting data sharing as a global public good, drawing on lessons learned from the COVID-19 pandemic [3].

Viral sequences and their associated metadata (e.g., the time the sequenced virus was isolated, the location of the individual from whom the virus was isolated) play a vital role in antiviral drug and vaccine development, drug resistance surveillance, and clinical management. In this manuscript, we review areas where sharing viral sequences and their metadata can further human immunodeficiency virus (HIV) drug resistance (HIVDR) research, impact HIV treatment guidelines, and enhance the clinical management of people living with HIV (PLWH).

Despite its benefits, public sharing of research data continues to be limited in numerous fields [4–8]. Given the extensive variety of data generated from research, funding agencies and journal editors often face uncertainty regarding types of data that should be shared for maximum public health benefit [9,10]. Consequently, it is crucial for key stakeholders to establish practical and domain-specific data-sharing standards. Experts in particular research fields are best suited to identify the most valuable, reusable, and shareable data in their domains without causing unnecessary burdens on researchers or posing risks to study participants. This project was initiated by the senior author in collaboration with frequent attendees of annual HIV drug resistance workshops who suggested soliciting input from additional global researchers involved in HIV antiretroviral (ARV) clinical trials and HIVDR surveillance.

Several reviews have outlined potential challenges to the sharing of published research data including legal issues related to ownership, incentives for researchers, technical issues (i.e., the types of data that should be shared and how they should be shared), and ethical challenges related to privacy [1,9–11]. This Policy Forum does not address the intricacy of legal issues as they may vary across regions and depend on the source of research funding. It instead focuses initially on the incentives for researchers indicating that HIVDR studies collectively form a vital knowledge base essential for interpreting HIVDR genotypic tests and monitoring global HIVDR trends, and then addresses the technical aspects of data sharing in HIVDR research and the unique ethical challenges associated with maintaining privacy in this context.

## Public health and clinical significance of HIVDR

In 2022, an estimated 29.8 million PLWH were receiving ARV treatment (ART) [12,13]. Approximately 10% to 15% of newly diagnosed PLWH have drug-resistant viruses [14], and an additional 2 million PLWH have likely experienced treatment failure, many of whom have also developed ARV drug-resistant viruses [14]. HIVDR has implications for ART strategies and for containing the HIV pandemic because the development of HIVDR leads to the requirement for ARV drugs that may be less effective, less well-tolerated, and more expensive than those used in first-line regimens. Transmitted HIVDR may also reduce the effectiveness of preexposure prophylaxis (PrEP), which is a critical component of global strategies for containing the spread of HIV [15,16].

HIVDR assessment for surveillance and for clinical purposes relies on genotypic sequencing and algorithms for predicting ARV drug susceptibility. However, interpreting genotypic HIVDR tests is challenging because there are many mutations associated with reduced ARV drug susceptibility, referred to as drug-resistance mutations (DRMs), and they differ in their effects on different drugs and often occur in complex patterns. Moreover, due to cross-resistance between drugs within the same ARV class, HIVDR interpretation should ideally be quantitative, to estimate the extent to which the mutations in a sequenced virus will compromise the virological response to an ARV drug or drug combination.

As new ARV drugs and data emerge, it is crucial to continuously update the approach for interpreting genotypic HIVDR profiles. Doing so ensures the optimization of individual patient care and enables the application of new HIVDR knowledge to the analysis of population-based surveys and clinical studies [17]. Such analyses also play a role in the updating of HIV treatment guidelines, including in regions where genotypic HIVDR tests are not routinely employed in clinical practice.

## HIVDR research data

HIV *pol* gene sequences obtained from ART-naïve persons offer insights into which positions in RT, protease, and integrase are conserved or polymorphic in the absence of selective ARV drug pressure. Sequences from ART-naïve individuals in regions with widespread ART utilization, when sampled appropriately, also help estimate the prevalence of transmitted HIVDR within a population. In such studies, transmitted HIVDR is defined as the presence of one or more nonpolymorphic DRMs that do not naturally arise in ART-naïve individuals [18].

Sequences from ART-experienced individuals yield important data on the viral genetic mutations responsible for resistance to specific ARV drugs and on the prevalence of acquired HIVDR associated with specific ART regimens. While data from population-based HIVDR surveillance are necessary to determine the prevalence of overall HIVDR and of specific DRMs in ART‐experienced populations, data from case reports and case series are often necessary to identify DRMs that emerge less commonly or that are associated with newly approved ARVs for which the incidence of emerging HIVDR in any single clinical trial or cohort is low. Importantly, it is also essential to analyze data from broad geographic regions, because viruses of different HIV subtypes differ in their propensity to develop specific DRMs under the influence of ARV drug selection pressure [17].

Once a comprehensive list of potential DRMs is identified, their biological and clinical significance must be assessed. Evaluating biological significance involves in vitro susceptibility testing of both site-directed mutants and clinical isolates containing 1 or more HIVDR mutations. For clinical isolates, susceptibility testing often requires examining multiple isolates with the same DRMs because the susceptibility associated with many DRMs can be influenced by background mutations present in clinical isolates. Evaluating clinical significance involves quantifying the impact of DRMs on the virological response to subsequent ART regimens.

## Gaps in HIVDR data sharing

Most journals mandate that sequences described in a published study should be submitted to the joint databases of GenBank, the DNA Data Bank of Japan, or the European Nucleotide Archive, which we refer to collectively as “GenBank” [19]. However, there has been less recognition of the importance of sequence metadata, which in the field of HIVDR also includes the ART history of the individual from whom the sequenced virus was obtained and, if available, in vitro susceptibility data and the virological response to subsequent ART regimens.

A recent meta-analysis of HIVDR data sharing examined 934 studies published between 2010 and 2019 [20]. Sequences were submitted to GenBank for 60% of HIVDR studies: 69% of ART-naïve studies, 47% of the ART-experienced studies, and 68% of the studies of children. Among the journals publishing more than 10 studies, sequence availability ranged from 8% to 87%. Sequences with linked ART histories were shared for only 20% of the studies of ART-experienced persons including from 37% of studies from sub-Saharan Africa, 22% from North America, and 8% from Europe [20].

There are even fewer sequences available from PLWH who have received newer ARV drugs. For example, in recent studies of PLWH receiving the recently approved ARVs doravirine and cabotegravir no sequences were submitted to GenBank [21,22]. Although the authors of these studies reported amino acid mutations they considered to be the most important DRMs, the absence of complete sequences (or lists of mutations) made it impossible to know whether unrecognized or accessory DRMs were also present.

The primary challenge to the availability of in vitro phenotypic susceptibility data is the cost of susceptibility testing. Phenotypic data sharing is typically not an issue, as authors of such studies generally make their data available in tables or figures.

The main challenge to the accessibility of virological response data is the additional effort involved in making such data available. This process typically requires the preparation of separate linked files containing sequences, ART histories, and virus levels. Furthermore, it may involve obtaining additional permissions to share this more complex data. Indeed, the individual patient-level ART history and virus load data associated with both clinical trials and cohort studies are rarely made publicly available.

## Privacy challenges associated with sharing HIV sequences and associated metadata

The analysis and sharing of viral sequences and associated epidemiological data hold public health value for understanding the transmission of HIV and other epidemic and pandemic viruses such as influenza, Ebola virus, and Severe Acute Respiratory Syndrome Coronavirus 2 (SARS-CoV-2) [23]. Phylogenetic analyses of viral sequences can characterize sequence diversity in a region and identify factors contributing to regional outbreaks [24]. When the demographics, behavioral histories, and stage of infection of individuals in densely sampled populations are known, it is possible to determine the characteristics and behaviors of those most likely to transmit or become infected by HIV, thus providing insights into optimal prevention measures and to identify emerging outbreaks. HIV *pol* sequences have often been used for these studies because they are widely available from routinely performed genotypic HIVDR testing.

In contrast to other viruses, sharing HIV sequences from densely sampled populations may pose unique risks because, in many countries and regions, HIV transmission is criminalized [25–27]. As a result, the consequences of a data breach or unintended identification of a study participant can be more detrimental to PLWH than to individuals infected with other viruses. In sufficiently densely sampled populations, it is likely that samples from transmission partners will have been sequenced and found to be highly similar. Nonetheless, without contact history data, it is not possible to determine whether individuals with highly similar sequences infected one another or were infected by a common source.

Public health agencies, which hold the mandate and authority to identify individuals and their contacts, have performed phylogenetic analyses to detect rapidly growing HIV clusters to identify communities affected by rapid HIV transmission for which prevention efforts are most needed [27,28]. In the US, the Centers for Disease Control and Prevention (CDC), which leads this effort, does not release these sequences to GenBank or other public repositories [27]. In contrast, phylogenetic research performed outside of public health agencies involves de-identified data and does not involve public health interventions at the individual level but may be useful for shaping population-based preventive measures. These researchers have often publicly shared a randomly selected subset of their sequences and offered restricted full access to other researchers through data use agreements [27].

Although HIV *pol* sequences are also used in phylogenetic studies of transmission dynamics, the density of sampling and types of metadata in phylogenetic studies differ from those of HIVDR studies. We therefore recommend that the ethical questions of sharing sequences from studies of transmission dynamics should be addressed separately by local human subjects review panels, authors, funding agencies, and communities of PLWH.

## HIVDR data sharing recommendations

The sidebar contains recommendations for sharing HIV *pol* sequences, ART histories, and virological outcome data for published HIVDR studies (Box 1). HIV *pol* sequences reported in HIVDR studies should be submitted to GenBank as this is the standard repository for genetic sequence data. Nucleotide sequences are critical for assessing sequence quality control and indicating the presence of synonymous and nonsynonymous variants and nucleotide mixtures [29]. For the purpose of furthering HIVDR research, nucleotide sequences also provide information on genetic barriers to resistance (i.e., number and type of nucleotide changes responsible for an amino acid mutation) and are essential for developing PCR primers and diagnostic reagents.

**Box 1. Recommendations for sharing HIV *pol* sequences and their associated metadata**

1. We recommend submitting HIV *pol* nucleotide sequences described in published studies to GenBank along with the following metadata:
  - Unique de-identified person ID for individuals with sequences in a given publication when there are multiple sequences from the same person. For example, samples may have been obtained at different times (e.g., before and after therapy) or from different specimen types (e.g., plasma, peripheral blood mononuclear cells). Sequences may also represent different clones from the same sample.
  - Specimen type (e.g., plasma virus, peripheral blood mononuclear cells, dried blood spots).
  - Method of cloning and sequencing (e.g., direct PCR dideoxyterminator sequencing, consensus of reads determined by a next-generation sequencing (NGS) technology, single genome sequencing).
  - Country in which the sequenced sample was obtained to enable regional estimates of HIVDR prevalence.
  - Year in which the sequenced sample was obtained to identify temporal trends in HIVDR prevalence.
  Notes: (1) If nucleotide sequences are not available or are deemed too sensitive to be made publicly available, the complete list of amino acid changes from a reference sequence should be made available as a supplementary data spreadsheet file attached to the publication in which the sequences are reported; (2) if nucleotide sequences are not available, the subtype should be provided; (3) for NGS consensus sequences, the mutation-detection threshold used to identify sub-consensus nucleotides should be specified.
2. We recommend that ART history data be included in publications or submitted to GenBank as a structured comment (https://www.ncbi.nlm.nih.gov/genbank/structuredcomment/). If the histories are complicated, they can be included as a supplementary data spreadsheet file. Ideally, the complete list of ART regimens received by an individual should be made available. However, the most recent ART regimen and all previously received ARVs would be potentially most useful.
3. Virological outcome data from clinical trials and cohort studies that provide insight into the clinical significance of HIVDR should be made publicly available in spreadsheets containing plasma HIV RNA levels temporally linked to HIV *pol* sequences and ART regimen changes. Course-grained data may be appropriate to reduce the risk of re-identification of de-identified data.

If sequencing was performed using a next-generation sequencing (NGS) technology, the inferred consensus sequence should be submitted along with the details of the analytical procedure used to determine the consensus sequence and to identify mutations detected at sub-consensus levels. Submission of the NGS files to the National Center for Biotechnology Information Sequence Read Archive is recommended for studies of intrahost evolution.

If authors are concerned that a data breach or the unintentional identification of persons in a study could place a person at risk of legal jeopardy because their sequence is very similar to that of another person in a study, the authors could translate the nucleotide sequence to amino acids before publicly sharing the sequence. Amino acid sequences contain less identifying information than nucleotide sequences yet are nearly as useful for HIVDR research.

If the ART histories in a study are not complicated (e.g., all samples were obtained from persons who were ART-naïve or who received just 1 or 2 ART regimens), they can be shared in the published paper and/or as a structured comment in GenBank. If the complete ART histories of persons in a study are not known or are complicated, sufficient treatment history for each individual to support the study’s findings should be shared as a supplementary data file. Finally, with the increasing use of PrEP globally, it has become important to document whether PLWH had received PrEP prior to HIV infection.

For clinical trials and cohort studies reporting the impact of baseline HIVDR on the virological response to an ART regimen, temporally linked sequences, ART regimens, and plasma HIV RNA levels should be shared either in a supplementary data file or in a research data repository that would satisfy the FAIR guiding principles for scientific data management—findable, accessible, interoperable, and reusable [30]. Even though participant data in such studies are de-identified, course-grained data should be provided where possible to mitigate the risk of patient re-identification.

Given sensitivities around patient identification, it is not necessary to share data on age, gender, ethnicity, or medical, social, and behavioral history because these factors have rarely been shown to influence which DRMs are selected by ART or to influence the biological or clinical effects of DRMs.

## Conclusions

Data on HIVDR hold significant value for HIV researchers, public health scientists, and PLWH. Nevertheless, while many studies collect HIVDR sequence and ART data, these data frequently remain inaccessible, thus limiting their inherent value to key stakeholders. We propose a comprehensive set of best practices to encourage and standardize HIVDR data sharing, which will maximize its potential in advancing HIV research, clinical care, and public health impact as well as safeguarding privacy. Although these recommendations are intended for authors of research studies, they will be most effective if journal editors incorporate them as publication prerequisites and funding agencies establish them as compliance benchmarks.

## Abbreviations

ARV: antiretroviral
ART: antiretroviral treatment
CDC: Centers for Disease Control and Prevention
DRM: drug-resistance mutation
HIV: human immunodeficiency virus
HIVDR: human immunodeficiency virus drug resistance
NGS: next-generation sequencing
NIH: National Institutes of Health
PLWH: people living with HIV
PrEP: preexposure prophylaxis
SARS-CoV-2: Severe Acute Respiratory Syndrome Coronavirus 2
WHO: World Health Organization

## References

[1] Contreras JL. NIH’s genomic data sharing policy: timing and tradeoffs. Trends Genet. 2015;31:55–57. doi: 10.1016/j.tig.2014.12.006 25620797

[2] NIH. NOT-OD-21-013: Final NIH Policy for Data Management and Sharing. 2020. Available from: https://grants.nih.gov/grants/guide/notice-files/NOT-OD-21-013.html. Accessed 2022 Aug 18.

[3] World Health Organization. Sharing and reuse of health-related data for research purposes: WHO policy and implementation guidance. 2022.

[4] Piwowar HA. Who Shares? Who Doesn’t? Factors Associated with Openly Archiving Raw Research Data. PLoS ONE. 2011;6:e18657. doi: 10.1371/journal.pone.0018657 21765886 PMC3135593

[5] Alsheikh-Ali AA, Qureshi W, Al-Mallah MH, Ioannidis JPA. Public availability of published research data in high-impact journals. PLoS ONE. 2011;6:e24357. doi: 10.1371/journal.pone.0024357 21915316 PMC3168487

[6] Stodden V, Seiler J, Ma Z. An empirical analysis of journal policy effectiveness for computational reproducibility. Proc Natl Acad Sci U S A. 2018;115:2584–2589. doi: 10.1073/pnas.1708290115 29531050 PMC5856507

[7] Danchev V, Min Y, Borghi J, Baiocchi M, Ioannidis JPA. Evaluation of Data Sharing After Implementation of the International Committee of Medical Journal Editors Data Sharing Statement Requirement. JAMA Netw Open. 2021;4:e2033972. doi: 10.1001/jamanetworkopen.2020.33972 33507256 PMC7844597

[8] Gabelica M, Bojčić R, Puljak L. Many researchers were not compliant with their published data sharing statement: a mixed-methods study. J Clin Epidemiol. 2022;150:33–41. doi: 10.1016/j.jclinepi.2022.05.019 35654271

[9] van Panhuis WG, Paul P, Emerson C, Grefenstette J, Wilder R, Herbst AJ, et al. A systematic review of barriers to data sharing in public health. BMC Public Health. 2014;14:1144. doi: 10.1186/1471-2458-14-1144 25377061 PMC4239377

[10] Anger M, Wendelborn C, Winkler EC, Schickhardt C. Neither carrots nor sticks? Challenges surrounding data sharing from the perspective of research funding agencies—A qualitative expert interview study. PLoS ONE. 2022;17:e0273259. doi: 10.1371/journal.pone.0273259 36070283 PMC9451069

[11] Gomes DGE, Pottier P, Crystal-Ornelas R, Hudgins EJ, Foroughirad V, Sánchez-Re LL, et al. Why don’t we share data and code? Perceived barriers and benefits to public archiving practices. Proc Biol Sci. 2022;289:20221113. doi: 10.1098/rspb.2022.1113 36416041 PMC9682438

[12] Bertagnolio S, Jordan MR, Giron A, Inzaule S. Epidemiology of HIV drug resistance in low- and middle-income countries and WHO global strategy to monitor its emergence. Curr Opin HIV AIDS. 2022;17:229–239. doi: 10.1097/COH.0000000000000743 35762378

[13] UNAIDS. 2023 UNAIDS GLOBAL AIDS UPDATE: Executive Summary the Path that Ends AIDS. 2023. Available from: https://unaids.org/sites/default/files/media_asset/2023-unaids-global-aids-update-summary_en.pdf.

[14] World Health Organization. HIV drug resistance report 2021. Geneva: World Health Organization; 2021. Available from: https://apps.who.int/iris/handle/10665/349340. Accessed 2022 Jun 22.

[15] Irungu EM, Baeten JM. PrEP rollout in Africa: status and opportunity. Nat Med. 2020;26:655–664. doi: 10.1038/s41591-020-0872-x 32405065

[16] To K, Lee S. A review of reported cases of HIV pre-exposure prophylaxis failure with resultant breakthrough HIV infections. HIV Med. 2021;22:75–82. doi: 10.1111/hiv.12989 33140556

[17] Günthard HF, Calvez V, Paredes R, Pillay D, Shafer RW, Wensing AM, et al. Human Immunodeficiency Virus Drug Resistance: 2018 Recommendations of the International Antiviral Society–USA Panel. Clin Infect Dis. 2019;68:177–187. doi: 10.1093/cid/ciy463 30052811 PMC6321850

[18] Bennett DE, Camacho RJ, Otelea D, Kuritzkes DR, Fleury H, Kiuchi M, et al. Drug resistance mutations for surveillance of transmitted HIV-1 drug-resistance: 2009 update. PLoS ONE. 2009;4:e4724. doi: 10.1371/journal.pone.0004724 19266092 PMC2648874

[19] Sayers EW, Cavanaugh M, Clark K, Pruitt KD, Sherry ST, Yankie L, et al. GenBank 2023 update. Nucleic Acids Res. 2023;51:D141–D144. doi: 10.1093/nar/gkac1012 36350640 PMC9825519

[20] Rhee S-Y, Kassaye SG, Jordan MR, Kouamou V, Katzenstein D, Shafer RW. Public availability of HIV-1 drug resistance sequence and treatment data: a systematic review. Lancet Microbe. 2022;3:e392–e398. doi: 10.1016/S2666-5247(21)00250-0 35544100 PMC9095989

[21] Martin EA, Lai M-T, Ngo W, Feng M, Graham D, Hazuda DJ, et al. Review of Doravirine Resistance Patterns Identified in Participants During Clinical Development. J Acquir Immune Defic Syndr. 2020;85:635–642. doi: 10.1097/QAI.0000000000002496 32925358 PMC7655028

[22] Overton ET, Richmond G, Rizzardini G, Jaeger H, Orrell C, Nagimova F, et al. Long-acting cabotegravir and rilpivirine dosed every 2 months in adults with HIV-1 infection (ATLAS-2M), 48-week results: a randomised, multicentre, open-label, phase 3b, non-inferiority study. Lancet. 2021;396:1994–2005. doi: 10.1016/S0140-6736(20)32666-0 33308425

[23] Yozwiak NL, Schaffner SF, Sabeti PC. Data sharing: Make outbreak research open access. Nature. 2015;518:477–479. doi: 10.1038/518477a 25719649

[24] France AM, Oster AM. The Promise and Complexities of Detecting and Monitoring HIV Transmission Clusters. J Infect Dis. 2020;221:1223–1225. doi: 10.1093/infdis/jiz177 31028707

[25] Mehta SR, Schairer C, Little S. Ethical issues in HIV phylogenetics and molecular epidemiology. Curr Opin HIV AIDS. 2019;14:221–226. doi: 10.1097/COH.0000000000000538 30946143 PMC6450547

[26] Coltart CEM, Hoppe A, Parker M, Dawson L, Amon JJ, Simwinga M, et al. Ethical considerations in global HIV phylogenetic research. Lancet HIV. 2018;5:e656–e666. doi: 10.1016/S2352-3018(18)30134-6 30174214 PMC7327184

[27] Dawson L, Benbow N, Fletcher FE, Kassaye S, Killelea A, Latham SR, et al. Addressing Ethical Challenges in US-Based HIV Phylogenetic Research. J Infect Dis. 2020;222:1997–2006. doi: 10.1093/infdis/jiaa107 32525980 PMC7661760

[28] Fauci AS, Redfield RR, Sigounas G, Weahkee MD, Giroir BP. Ending the HIV Epidemic: A Plan for the United States. JAMA. 2019;321:844–845. doi: 10.1001/jama.2019.1343 30730529

[29] Learn GH, Korber BT, Foley B, Hahn BH, Wolinsky SM, Mullins JI. Maintaining the integrity of human immunodeficiency virus sequence databases. J Virol. 1996;70:5720–5730. doi: 10.1128/JVI.70.8.5720-5730.1996 8764096 PMC190542

[30] Wilkinson MD, Dumontier M, Aalbersberg IJJ, Appleton G, Axton M, Baak A, et al. The FAIR Guiding Principles for scientific data management and stewardship. Sci Data. 2016;3:160018. doi: 10.1038/sdata.2016.18 26978244 PMC4792175

